# A narrow QRS gallop rhythm

**DOI:** 10.1007/s12471-015-0653-1

**Published:** 2015-01-28

**Authors:** Daniel Mol, Andrew Tjon Joek Tjien, Jonas S.S.G. de Jong

**Affiliations:** Department of cardiology and cardiac surgery, Onze Lieve Vrouwe Gasthuis, Oosterpark 9, 1091 AC Amsterdam, The Netherlands

**Fig 1:**
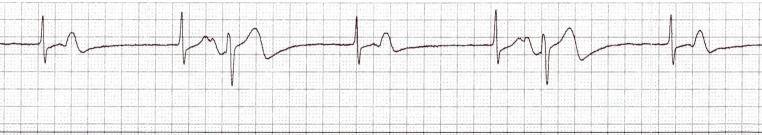
ECG recording during surgical pulmonary vein isolation

A 59-year-old male patient was referred to our hospital for treatment of paroxysmal atrial fibrillation since 8 years, with frequent presentations to the emergency department for cardioversion. Previously, post-cardioversion sinus bradycardia was observed. After extensive discussion of the treatment options, the patient opted for a minimally invasive surgical pulmonary vein isolation. The ECG shown was recorded during ablation of the left-sided pulmonary veins (Fig. 1).

Question

What is the mechanism of this trigeminy rhythm?

You will find the answer elsewhere in this issue.

